# Clinical and EEG characteristics of Juvenile Myoclonic Epilepsy

**DOI:** 10.12669/pjms.301.4465

**Published:** 2014

**Authors:** Shahnaz, Khalid Sher, Rukhsana Abdul Sattar

**Affiliations:** 1 Dr. Shahnaz, FCPS, Senior Registrar, Department of Neurology, Jinnah Postgraduate Medical Centre, Karachi, Pakistan.; 2 Dr. Khalid Sher, FCPS, MD,Assistant Professor, Department of Neurology, Jinnah Postgraduate Medical Centre, Karachi, Pakistan.; 3 Prof. Rukhsana Abdul Sattar, FCPS, Department of Medicine, Jinnah Postgraduate Medical Centre, Karachi, Pakistan.

**Keywords:** Clinical, Electroencephalography, Juvenile, Myoclonic Epilepsy

## Abstract

***Objective*** *:* To determine the clinical and electroencephalographic characteristics of patients with Juvenile Myoclonic Epilepsy (JME).

***Methods:*** In this descriptive case series study, 60 patients of Juvenile myoclonic epilepsy (JME) were included. After detailed history clinical examination, Electroencephalography (EEG) with standard protocol was performed in all patients and was analyzed by a neurologist.

***Results:*** Out of 60 patients, 26 (43.3%) were males and 34 (56.6%) were females. Mean age at the onset of myoclonic jerks (MJ) and generalized tonic clonic seizures (GTCS) was 13.7 ± 2.12 years and 14.15 ± 1.79 years respectively. Average delay in the diagnosis was 5.2 years. Myoclonic jerks (MJ) were present in all patients, GTCS in 52 (86.6%), and absence seizures in 8 (13.33%) patients. 6 (10%) had only Myoclonic Jerks. First seizure type was MJ in 52 (86.6%) and absence in 8 (13.3%). Most common precipitating factors were sleep deprivation in 80% and fatigue in 66.6%. Family history for epilepsy was positive in 20%. Diagnosis by referring physicians was JME in only 6 (10%) patients. EEG was abnormal in 42 patients (70%) showing generalized , 4- to 6-Hz polyspike and wave in 27 (45%), generalized single spike/ sharp waves in 7 patients (11.6%), 8 (13.3%) patients had 3-Hz spike-and-wave (SW) activity in addition to the polyspike-and-wave (PSW) pattern. Independent focal EEG abnormalities were noted in 12 patients (20%).

***Conclusion:*** Many of our patients were misdiagnosed by the referring physicians and were prescribed inappropriate antiepileptic drugs. Factors causing misdiagnosis were failure to elicit history of myoclonic jerks, misinterpreting myoclonic jerks as partial seizures and misinterpretation of EEG abnormalities.

## INTRODUCTION

Epilepsy prevalence in Pakistan is 1%.^1^ Janz described Juvenile Myoclonic Epilepsy (JME) for the first time in 1957.^2^ Juvenile myoclonic epilepsy (JME) is an idiopathic generalized epileptic syndrome with age related onset.^3^ The prevalence of JME among other adult and adolescence onset epilepsies is between 4-11%.^4^ JME begins in the second decade of life with myoclonic jerks (MJ) and in most of patients generalized tonic clonic seizures (GTCS) are found. Absence seizures can be present in around 1/3 of patients. Seizures are precipitated by sleep deprivation, fatigue, alcohol intake and flashing lights.^4^ As mentioned in different series epilepsy is found at a ratio of 27.3-44.2 % in the families of patients.^4^^,^^5^

Despite the distinctive clinical and electroencephalographic features known for five decades, juvenile myoclonic epilepsy (JME) is frequently unrecognized and misdiagnosed in both developed and developing countries,^4^ mainly because the early morning myoclonic seizures are not mentioned by the patients until specifically asked and also due to misinterpretation of EEG findings. Myoclonic jerks reported as unilateral, nocturnal generalized tonic-clonic seizures and focal EEG abnormalities are other factors contributing to misdiagnosis.^6^

This study was conducted to determine the clinical and electroencephalographic (EEG) characteristics of patients with JME at a tertiary care hospital in Karachi.

## METHODS

This was a descriptive case series study, conducted at the dept of Neurology, Jinnah Postgraduate Medical centre (JPMC), Karachi from 1^st^ February 2010 to 31^st^ Dec 2011. The study was approved by the ethical committee, JPMC. Sixty patients, regardless of age and gender, diagnosed as Juvenile myoclonic epilepsy by a Neurologist were included in this study. The inclusion criteria for JME were (a) unequivocal clinical evidence of generalized seizures with myoclonic jerks usually on awakening; (b) no evidence of focal neurological deficit or cognitive decline on clinical examination and (c) normal brain imaging when performed.

We excluded those patients with: (a) clinical or EEG evidence of myoclonic jerks secondary to hypoxia, metabolic disease or other structural brain abnormalities; (b) other generalized seizures without firm evidence of myoclonic jerks; and (c) EEG abnormalities, but no clinical evidence of any type of seizures and d) family history of progressive myoclonic epilepsy. 

Informed consent was taken from the patients. Detailed history was taken and detailed clinical examination was done in all patients. EEG with standard protocol was performed in all patients. Intermittent photic stimulation was carried out in all patients with eyes closed. EEG findings were analyzed by a neurologist. Those patients with normal or borderline initial EEG underwent sleep deprived EEG. The antiepileptic therapy that patients were already taking was modified and continued. Data was recorded on a pre-designed pro forma and was analyzed using SPSS v 18. Percentages, mean and median were calculated for different variables.

## RESULTS

Our study included 60 patients of JME, 26 (43.3%) were males and 34 (56.6%) were females. Mean age of patients was 20.35 ±4.94 years. Mean age at the onset of myoclonic seizures, GTCS and absence seizures was 13.7 ± 2.12, 14.15 ± 1.79 and 11.5 ± 3 years respectively. Types of seizures included myoclonic jerks (MJ) in all patients, GTCS in 52 (86.6%), and absence seizures in 8 (13.3%) patients. Six (10%) patients had only Myoclonic seizures (Table-I).

Early onset absence seizures were found in 2 (3.33%) and late onset in 6 (10%) patients. First seizure type was Myoclonic jerks (MJ) in 52 (86.6%), absence in 8 (13.3%) and GTCS in none. Latest age for MJ was 26, for absence 16, and for GTCS was 27 years.

Myoclonic Jerks involved both upper extremities symmetrically in 51 (85%) patients, 8 (13.3%) had asymmetrical myoclonic jerks in upper extremities, and one patient had involvement of lower extremities also.

The precipitating factors included sleep deprivation in 48 patients (80%), fatigue in 40 (66.6%), stress in 12 (20%), menstruation in 7 (20.5% of female patients), watching television and video games in 4 patients (6.66%). Six patients (10%) didn’t have any particular precipitating factor. Family history for epilepsy was positive in 12 (20%) patients, 4 in first degree relatives and 8 in second degree relatives.

Diagnosis by referring physicians was JME in only 6 (10%) patients, seizure disorder in 6 patients (10%), epilepsy in 18 (30%) , GTCS in 22 (36.6%), and partial seizures in 8 (13.3%) patients. Average delay in the diagnosis was 5.22 years (ranged from 4-10 years). Antiepileptics which were prescribed included carbamazepine in 30%, phenobarbitone in 20%, valproate in 35%, phenytoin in 8% and other antiepileptics in 7%. There were 36 (60%) patients who were on polytherapy including phenobarbitone, phenytoin, and Carbamazepine.

Regarding EEG findings, 30% (18) had normal EEG, EEG was abnormal in 70% (42). Among patients with abnormal EEG, there was generalized , 4- to 6-Hz polyspike and wave in 27 (45%), generalized single spikes/ sharp waves in 7 patients(11.6%), 8 (13.3%) patients had 3-Hz spike-and-wave (SW) activity in addition to the polyspike-and-wave (PSW) pattern. Intermittent photic stimulation precipitated spike and wave pattern in 8 patients (13.3%), more so in females than males (6 females, 2 males). Asymmetry of SW/PSW discharges was found in 6 (10%). Focal EEG abnormalities were noted in 12 (20%) patients which included focal spikes before or after the generalized Spike and waves (SW) in 4 (6.66%) patients, asymmetry of SW/PSW discharges in 6 (10%) patients, independent focal spikes/SW activity in 4 (6.66%) and focal slowing in 3 (5%) patients. Five (8.33%) patients had more than one focal EEG abnormalities.

**Table-I:** Frequency of different seizure types in JME

*Type of seizure*	*n (%)*
Myoclonic	60 (100%)
GTCS	52 (86.6%)
Absence	8 (13.3%)
Absence + Myoclonic	2 (3.3%)
Absence + myoclonic + GTCS	6 (10%)
Myoclonic + GTCS	46 (76.6%)
Myoclonic seizures alone	6 (10%)

## DISCUSSION

Our study showed slight female predominance (56.6% females) in JME patients. Gender distribution is considered to be equal but few studies have shown female preponderance.^3^^,^^5^

Juvenile myoclonic epilepsy (JME) begins between the ages of 8-26 years.^5^ Majority of the patients have their first seizures between the ages of 12-18 years.^7^ In our series, mean age of the patients was 20.35 ±4.94 years. Mean age at the onset of myoclonic seizures & GTCS was 13.7 ± 2.12 years & 14.15 ± 1.79 years respectively. Absence seizures were found in 13.3% patients in our study compared to 35% reported among patients belonging to different ethnic groups.^8^

In our series, the earliest age of onset of Myoclonic seizures and generalized tonic clonic seizures (GTCS) was ten years and absence seizures at 8 years. Before starting treatment, myoclonic seizures in 52 (86.6%) patients and GTCS in 45 (75%) patients occurred most often immediately after awakening. This circadian rhythm was observed in other studies also.^5^^,^^8^^,^^9^ In those patients who had absence seizures, these always preceded myoclonic seizures and GTCS. Similar observation was made in other studies also.^9^^,^^10^

Commonest precipitating factors were found to be sleep deprivation, followed by fatigue and stress. Similar observation was made in other studies done in India^4^, England^8^ and Iran.^9^ Menstruation precipitated seizures in 20% of women which was slightly higher than reported in few other studies.^4^^,^^9^ Alcohol wasn’t found to be an important precipitating factor as its consumption is limited in our society.

Family history in our series was present in 12 cases, 4 in first degree relatives and 8 in second degree relatives. Four of them carried the diagnosis of JME. However we couldn’t confirm the exact type of epilepsy in rest of the affected family members because they were unable to visit our outpatient clinic for clinical assessment. In different studies family history was present in 27.3-44.2%.^5^^,^^10^

Diagnosis of JME was made in only 6 (10%) patients by the referring physicians. Average delay in the diagnosis was 5.2 years (maximum 9 years). Delay in the diagnosis has been observed in many studies, ranging from a mean of 6.8 to 15.0 years, both from developed and developing countries.^10^ Majority of the clinicians failed to elicit the history of myoclonic jerks and interpret EEG findings. Myoclonic jerks are usually not mentioned by the patients until specifically asked, they consider it unimportant and most of the time they don’t seek medical advice for it and most of them consider it as clumsiness. Predominantly unilateral myoclonic seizures give the false impression of partial seizures. Many of our patients were taking those antiepileptics which can worsen myoclonic seizures e.g. carbamazepine, phenobarbitone and phenytoin.

The reported incidence of interictal EEG abnormalities is 76-88%.^8^^,^^11^ Most common EEG findings in our patients were generalized 4- to 6-Hz polyspike and wave in 45% (27) followed by generalized single spikes/ sharp waves in 11.6% (7 p). Subtle focal EEG abnormalities may be seen in 30%–50% of JME patients.^12^ In our series, independent focal EEG abnormalities were noted in 12 (20%) patients. Asymmetry of SW/PSW discharges was found in 6 (10%).

The photoconvulsive response has been reported in one-third of JME patients but it is variable according to the methodology used.^11^^,^^13^ In our study it was observed in 11 (18.33%) patients. A low prevalence of photoparoxysmal response has been observed in African and Asian patients.^4^

## CONCLUSION

Most of the clinical and EEG features of our series were in accordance with the literature. Many of our patients were misdiagnosed by the referring physicians and were prescribed inappropriate antiepileptic drugs. Factors causing misdiagnosis were failure to elicit history of myoclonic jerks, misinterpreting myoclonic jerks as partial seizures and misinterpretation of EEG abnormalities. The diagnosis of JME depends on the proper background knowledge of the syndrome, eliciting history of and properly interpreting the myoclonic seizures. EEG should be used as an ancillary diagnostic tool.

## Authors Contribution:

**S** designed the study, did data collection, statistical analysis & prepared the manuscript.

**KS** did data collection and editing of manuscript.

**RAS** did review and final approval of manuscript.
